# Speech Comprehension Difficulties in Chronic Tinnitus and Its Relation to Hyperacusis

**DOI:** 10.3389/fnagi.2016.00293

**Published:** 2016-12-15

**Authors:** Veronika Vielsmeier, Peter M. Kreuzer, Frank Haubner, Thomas Steffens, Philipp R. O. Semmler, Tobias Kleinjung, Winfried Schlee, Berthold Langguth, Martin Schecklmann

**Affiliations:** ^1^Department of Otorhinolaryngology, University of RegensburgRegensburg, Germany; ^2^Interdisciplinary Tinnitus Center of the University of RegensburgRegensburg, Germany; ^3^Department of Psychiatry and Psychotherapy, University of RegensburgRegensburg, Germany; ^4^Department of Otorhinolaryngology, University of ZurichSwitzerland

**Keywords:** chronic tinnitus, speech perception problems, Goettingen sentence test, hearing loss, hyperacusis

## Abstract

**Objective:** Many tinnitus patients complain about difficulties regarding speech comprehension. In spite of the high clinical relevance little is known about underlying mechanisms and predisposing factors. Here, we performed an exploratory investigation in a large sample of tinnitus patients to (1) estimate the prevalence of speech comprehension difficulties among tinnitus patients, to (2) compare subjective reports of speech comprehension difficulties with behavioral measurements in a standardized speech comprehension test and to (3) explore underlying mechanisms by analyzing the relationship between speech comprehension difficulties and peripheral hearing function (pure tone audiogram), as well as with co-morbid hyperacusis as a central auditory processing disorder.

**Subjects and Methods:** Speech comprehension was assessed in 361 tinnitus patients presenting between 07/2012 and 08/2014 at the Interdisciplinary Tinnitus Clinic at the University of Regensburg. The assessment included standard audiological assessments (pure tone audiometry, tinnitus pitch, and loudness matching), the Goettingen sentence test (in quiet) for speech audiometric evaluation, two questions about hyperacusis, and two questions about speech comprehension in quiet and noisy environments (“How would you rate your ability to understand speech?”; “How would you rate your ability to follow a conversation when multiple people are speaking simultaneously?”).

**Results:** Subjectively-reported speech comprehension deficits are frequent among tinnitus patients, especially in noisy environments (cocktail party situation). 74.2% of all investigated patients showed disturbed speech comprehension (indicated by values above 21.5 dB SPL in the Goettingen sentence test). Subjective speech comprehension complaints (both for general and in noisy environment) were correlated with hearing level and with audiologically-assessed speech comprehension ability. In contrast, co-morbid hyperacusis was only correlated with speech comprehension difficulties in noisy environments, but not with speech comprehension difficulties in general.

**Conclusion:** Speech comprehension deficits are frequent among tinnitus patients. Whereas speech comprehension deficits in quiet environments are primarily due to peripheral hearing loss, speech comprehension deficits in noisy environments are related to both peripheral hearing loss and dysfunctional central auditory processing. Disturbed speech comprehension in noisy environments might be modulated by a central inhibitory deficit. In addition, attentional and cognitive aspects may play a role.

## Introduction

Subjective tinnitus is the perception of sound in the absence of a corresponding acoustic signal (Baguley et al., 2013). Hearing loss is a recognized risk factor for tinnitus (Hoffmann and Reed, 2004). It is assumed that tinnitus is generated by plastic changes in the central nervous system as a reaction to reduced auditory input similar to mechanisms associated with phantom perceptions after limb amputation (De Ridder et al., 2011). Accordingly, the majority of tinnitus patients show elevated hearing thresholds in the standard pure tone audiogram (Norena et al., 2002). And many tinnitus patients with normal or nearly normal hearing thresholds in the pure tone audiogram exhibit cochlear damage (Shore et al., 2016). Hair cell loss at frequencies between the tested frequencies (“dead regions”) (Weisz et al., 2006) or at high frequencies (Vielsmeier et al., 2015) have been observed in tinnitus patients with normal standard audiograms. There is also evidence for damage of high-threshold auditory nerve fibers in tinnitus patients with normal standard audiograms (Schaette and McAlpine, 2011). Speech comprehension difficulties are frequently reported by tinnitus patients (Tyler and Baker, 1983; Newman et al., 1994), even by those with normal pure tone audiograms. Particular difficulties in following a conversation are reported in situations when multiple people are speaking simultaneously, e.g., in a classical “cocktail party situation” (Jones and Litovsky, 2008).

Speech comprehension difficulties are for many tinnitus patients among the leading causes for their tinnitus-related handicap and therefore clinically, highly relevant. In contrast, knowledge about prevalence, assessment, pathogenetic mechanisms, and management of speech comprehension impairment in tinnitus patients is still very limited. Only few studies have addressed specifically impaired speech comprehension abilities in tinnitus patients. An early study demonstrated that 13 out of 25 normal hearing patients with tinnitus have deficits in at least one out of several conducted speech comprehension tests (Goldstein and Shulman, 1999). The investigation of a large sample of 495 workers who suffered from tinnitus showed speech intelligibility deficits as compared to workers without tinnitus (Soalheiro et al., 2012). A small study investigating 20 Chinese speaking patients with tinnitus revealed that speech comprehension was impaired as compared to a control group, particularly in noisy environments (Huang et al., 2007). Another study revealed that masking of tinnitus by sound improved speech comprehension (Ryu et al., 2012). An improvement of speech comprehension in noise was also observed in patients with unilateral cochlear implants when tinnitus loudness was reduced by activation of cochlear implants (Mertens et al., 2013a) or in patients whose tinnitus was successfully suppressed by rTMS treatment (Barwood et al., 2013). Wearing hearing aids over a time of 3 months showed amelioration of the performance in two speech comprehension tests for elderly patients (Araujo and Iório, 2016). Speech comprehension was worse in patients with tinnitus at the beginning of the trial, improved in patients with and without tinnitus during the trial, but speech comprehension abilities remained reduced in all evaluated parameters at the end of the treatment.

These findings suggest that tinnitus and impaired speech comprehension in noise share a common pathophysiological substrate in the central auditory pathways, a notion supported by a recent study demonstrating that ears with and without tinnitus did not differ in auditory spectral and temporal resolution abilities, but in speech comprehension in noise (Moon et al., 2015). It seems also likely that speech comprehension deficits among tinnitus patients are not solely explainable by cochlear damage, as speech comprehension deficits are also reported by patients with normal audiograms.

One of the most common comorbidities with a prevalence of 40–55% in chronic tinnitus is hyperacusis (Schecklmann et al., 2014). Hyperacusis is in general defined as abnormal intolerance to sounds. For this work we define hyperacusis as stated in the Tinnitus Sample Case History Questionnaire (TSCHQ) from the TRI (Tinnitus Research Initiative) database (refer to the methods; Schecklmann et al., 2014, 2015). Two studies investigated the relationship between hyperacusis and speech comprehension: Ten patients with auditory processing disorder, but normal audiogram, showed elevated hyperacusis scores and worse transient evoked otoacoustic emission suppression test results in contrast to 12 age-matched controls (Spyridakou et al., 2012). Strong correlations were found for a speech in babble test targeting the right ear and self-described speech in noise understanding, and also for a transient evoked otoacoustic emission suppression test targeting the right ear and hyperacusis. Nineteen normal-hearing patients with tinnitus and hyperacusis showed similar performance in speech comprehension in silence but lower performance in a communication scenario in contrast to 23 normal hearing subjects without hearing complaints (Hennig et al., 2012). The authors speculated that the common pathophysiologic substrate of tinnitus, hyperacusis, and speech comprehension (in noise) may be a dysfunction in the medial olivary cochlear system. This is an efferent inhibitory system from the primary auditory cortex along the auditory pathway to the outer and inner hair cells of the cochlea with the function to filter out irrelevant noise (Harkrider and Bowers, 2009).

In summary, the available data suggest that both peripheral and central auditory dysfunction is involved in speech comprehension difficulties in tinnitus (and hyperacusis) patients. However, most available data come from small and heterogeneous samples and there is only very limited information about how many tinnitus patients suffer from speech comprehension difficulties in quiet and in noisy environments, and how these communication difficulties relate to audiometric findings and to tinnitus characteristics. Moreover no established standard exists for the clinical assessment of speech comprehension difficulties. The mechanisms of this phenomenon are still incompletely understood and no specific evidence-based treatments exist.

The primary aim of the present work was to investigate how many patients with tinnitus suffer from subjective speech comprehension in quiet and in noisy environments. The second aim was to investigate how subjective impairments in speech comprehension relate to audiological findings in the standard audiogram and in a validated sentence comprehension test. The third aim was to explore the relationship between speech comprehension difficulties and co-morbid hyperacusis.

## Materials and methods

### Subjects/sample

All patients presenting with subjective chronic tinnitus at the Interdisciplinary Tinnitus Center at the University of Regensburg (Regensburg, Germany, a tertiary referral center) between July, 1st 2012 and August, 31th 2014 were invited to participate in this exploratory prospective study.

### Assessment

All patients completed various tinnitus questionnaires including the Tinnitus Sample Case History Questionnaire (TSCHQ; Langguth et al., 2007) and the German version of the Tinnitus Questionnaire (TQ; Goebel and Hiller, 1994; range: 0–84 with higher scores presenting higher distress), underwent microscopy of the ear and received an audiological examination including pure tone audiometry (125–8000 Hz), stapedius reflex testing, and tympanometry.

In addition to this standard assessment, speech comprehension was prospectively assessed by a specific German sentence test [“Goettingen Satztest” (Kollmeier and Wesselkamp, 1997)] and by asking two subjective questions about speech comprehension impairment.

The Goettingen sentence test was chosen as it reflects a realistic comprehension situation and has a high ecological validity (Kollmeier and Wesselkamp, 1997; Arweiler-Harbeck et al., 2011; May-Mederake and Shehata-Dieler, 2013). This test was performed in silence via air conduction by using headphones. A total of 20 sentences were presented starting at 35 dB SPL. Based on the results given by the patient the loudness was elevated or reduced accordingly in the upcoming sentences. The result is expressed as the 50% perception-threshold in dB and can range from 0 to 100 dB SPL.

Subjective speech comprehension impairment was assessed by the following two questions: “How would you rate your ability to understand speech? (German: Wieviele Probleme haben Sie, Sprache zu verstehen?)” and “How would you rate your ability to follow a conversation when multiple people are speaking simultaneously (e.g., in restaurant situation)? [German: Wieviele Probleme haben Sie, Gesprächen zu folgen, wenn mehrere Personen gleichzeitig sprechen (z.B. in einer Gaststätte)?]” on a scale ranging between 0 and 5 points (0, no problems; 1, minimal problems; 2, minor problems; 3, moderate problems; 4, significant problems; 5, massive problems; German: 0, keine Probleme; 1, sehr wenig Probleme; 2, wenig Probleme; 3, mäßig; 4, starke Probleme; 5, sehr starke Probleme). Patients gave written informed consent that data were gathered and analyzed for the Tinnitus Research Initiative Database which was approved by the Ethics Committee of the University Hospital of Regensburg (Germany; reference number 08/046).

For descriptive analyses, “normal” was defined by the cut-off criterion that the hearing thresholds at all frequencies measured in the standard pure tone audiogram (125, 250, 500 Hz, 1, 2, 4, 6, 8 kHz) were equal or below 20 dB hearing loss. The mean value over all frequencies (125, 250, 500 Hz, 1, 2, 4, 6, 8 kHz) and both ears was calculated for each patient. Patients with normal or disturbed speech comprehension in accordance to the Goettingen sentence test were determined by the cut-off criterion ≤ 21.5 dB SPL.

Moreover we explored whether tolerability of loud sounds (hyperacusis) was related to subjectively perceived speech comprehension deficits. For this purpose, we used two questions [“Do you have a problem tolerating sounds because they often seem much too loud? That is, do you often find sounds too loud or hurtful, which other people around you find quite comfortable?” (answers: never, rarely, sometimes, usually, always; rated with 1, 2, 3, 4, 5, respectively); “Do sounds cause you pain or physical discomfort?” (answers: no, yes, I don't know; rated with 0, 1, 2, respectively)], that have recently been validated as useful screening questions for hyperacusis (Schecklmann et al., 2015). Notably, these questions focus particularly on symptoms of fear/pain-related hyperacusis and do not diagnosis hyperacusis comprehensively.

Data were collected within the framework of the Tinnitus Research Initiative Database (Landgrebe et al., 2010).

### Statistical analysis

For statistical analyses, we initially present descriptive data and the number of patients with speech comprehension problems. Speech comprehension ratings are also shown compared to normal values for the audiogram and Goettingen test results. To evaluate possible factors contributing to subjective speech comprehension deficits (dependent variables) we analyzed possible associations by using Pearson correlation coefficients for metric variables (hearing level, Goettingen Satztest, and hyperacusis question 1 with speech comprehension questions 1 and 2) and Student *t*-tests for independent samples for dichotomous variables (patients with yes vs. no answers in hyperacusis question 2 with respect to speech comprehension questions 1 and 2). For these analyses, we firstly used the average hearing level and the Goettingen test score. We assumed that these audiologic variables were related to the subjectively perceived deficit in speech comprehension. To assess the influence of sound tolerability on speech comprehension we correlated the answers for the question “Do you have a problem tolerating sounds because they often seem much too loud? That is, do you often find sounds too loud or hurtful, which other people around you find quite comfortable?” and “Do sounds cause you pain or physical discomfort?” with the speech comprehension scores and compared the speech comprehension scores between patients who experienced physical discomfort or pain to sounds and those who did not (Table 1). All association analyses were repeated with the Tinnitus severity (Tinnitus Questionnaire Score) as co-variate by using partial correlations for the Pearson correlations and analyses of covariance for the Student *t*-test. Spearmen correlations revealed the same results. As partial correlations are only possible for parametric tests we provide results of the Pearson correlations. The significance threshold was set to 5%. No corrections for multiple comparisons were performed because of the explorative character of the study. Strength of correlations was indicated as small (*r* = 0.1), medium (*r* = 0.3), and high (*r* = 0.5). Effect sizes for Student *t*-tests were indicated by Cohen's d with 0.2 as small, 0.5 as medium and 0.8 as large effect size. Statistical analyses were performed with SPSS (SPSS Inc., USA, version 22).

**Table 1:** **(A) Association of variables of audiometry, Goettingen sentence test, and hyperacusis with subjective speech comprehension**.

	**General speech comprehension problems (0–5)**	**Speech comprehension problems in noisy environment (0–5)**
Average hearing loss (dB HL) (*n* = 361)	*r* = 0.474; *p* < 0.001	*r* = 0.517; *p* < 0.001
Goettingen sentence test (dB SPL) (*n* = 361)	*r* = 0.351; *p* < 0.001	*r* = 0.387; *p* < 0.001
Do you have a problem tolerating sounds because they often seem much too loud? That is, do you often find too loud or hurtful sounds which other people around you find quite comfortable? (*n* = 349)	*r* = 0.071; *p* = 0.188	*r* = 0.268; *p* < 0.001
Do sounds cause you pain or physical discomfort? (yes: *n* = 188; no = 126)	yes: 0.96 ± 1.25; no: 0.83 ± 1.17; *t* = 0.982; *df* = 312; *p* = 0.327; *d* = 0.107	yes: 2.66 ± 1.54; no: 2.10 ± 1.62; *t* = 3.140; *df* = 312; *p* = 0.002; *d* = 0.354

*r, Pearson correlations coefficient; t, Student t-test; xx ± xx, mean ± sd*.

## Results

**Figure 1:**
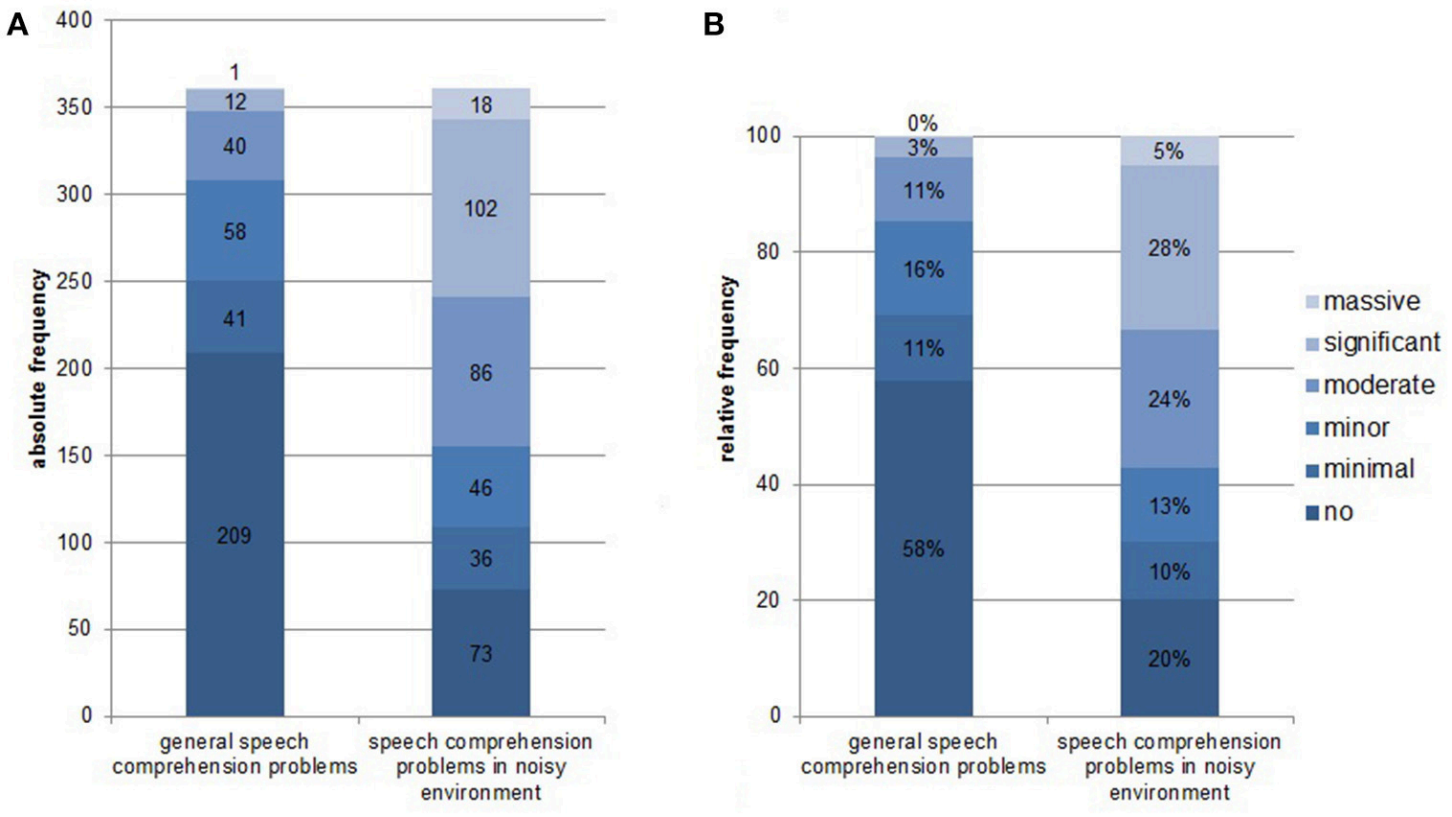
**Distribution of answers from the speech comprehension questions [“How would you rate your ability to understand speech?” and “How would you rate your ability to follow a conversation when multiple people are speaking simultaneously (e.g., in restaurant situation)? on a scale ranging between 0, no problems; 1, minimal problems; 2, minor problems; 3, moderate problems; 4, significant problems; to 5, massive problems]. (A)** Absolute frequency of distribution; **(B)** Relative frequency of distribution.

**Figure 2:**
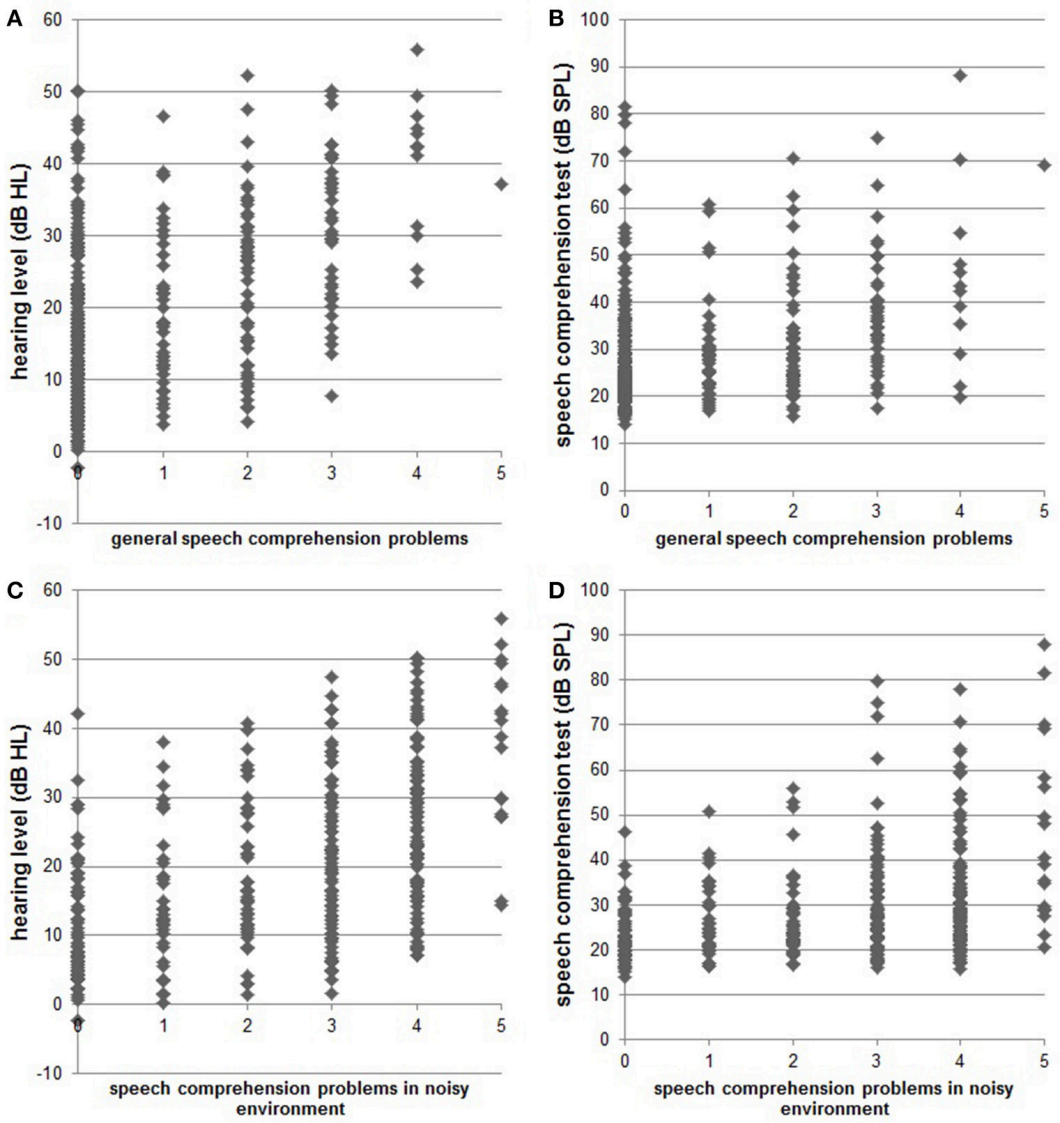
**Scatter plots indicate single subject data for hearing status [pure tone audiogram; (A)**: *r* = 0.474; **(C)**: *r* = 0.517] and speech comprehension status [results from Goettingen sentence test; **(B)**: *r* = 0.351, **(D)**: *r* = 0.387] compared to the subjective speech comprehension report in quiet and in noisy environments, respectively.

Complete data sets (audiogram, Goettingen sentence test, speech comprehension questions) were available from 361 patients. One hundred and thirty-one (36.3%) were female, 51 (14.1%) reported right-sided tinnitus, 92 (25.5%) left-sided tinnitus, and 216 (59.8%) tinnitus in both ears or within the head. Age was 52.4 ± 12.5 (mean ± SD) years, tinnitus distress was 39.8 ± 17.3 as indicated by the tinnitus questionnaire (Hiller and Goebel, 1992; Goebel and Hiller, 1994; Hiller et al., 1994), and tinnitus duration was 93.8 ± 97.0 months. 296 (82%) patients had hearing loss (>20 dB HL in one or more frequencies of the standard audiogram) and 268 (74.2%) showed disturbed speech comprehension as indicated by values above 21.5 dB SPL in the Goettingen sentence test. One hundred and fifty-two (42.1%) patients reported problems with speech comprehension in general (score of at least one in question 1) and 288 (79.8%) problems with speech comprehension when multiple people were speaking simultaneously (speech comprehension in group conversation or noisy environment; score of at least one in question 2). For detailed distribution of answers to the two questions see Figure 1. As can be seen in Figure 2 there is a quite good overlap of audiometry results or results from the Goettingen sentence test with subjective ratings of speech comprehension. This is also evident by correlating these variables showing significant positive correlations of the mean audiogram and the comprehension test with subjective speech comprehension in general and in noisy environment with medium to high correlations coefficients (Table 1A).

Moreover we assessed the influence of co-morbid hyperacusis (low tolerability of loud sounds) on speech comprehension (Table 1A). Patients with hyperacusis (Schecklmann et al., 2015) showed an association with speech comprehension problems in noisy environments (negligible effects sizes), but not in quiet environments (small effect sizes correlation coefficient). For the first hyperacusis question, patients reporting greater problems with sound tolerance showed greater problems with speech comprehension in noisy environments and vice versa. For hyperacusis question two, patients with discomfort to sounds report significant higher speech comprehension deficits in noisy environments in contrast to patients without discomfort to sounds.

All correlations in Table 1A were also repeated with the Tinnitus severity (Tinnitus Questionnaire Score) as co-variate. Apart from the correlation between the categorical hyperacousis question “Do sounds cause you pain or physical discomfort?” and the speech comprehension in noise score, which lost significance after inclusion of the TQ score as co-variate, results remained unchanged (Table 1B), indicating that the main findings are not driven by global tinnitus severity.

**Table 1:** **(B) Association of variables of audiometry, Goettingen sentence test, and hyperacusis with subjective speech comprehension with the tinnitus questionnaire as covariate**.

	**General speech comprehension problems (0–5)**	**Speech comprehension problems in noisy environment (0–5)**
Average hearing loss (dB HL) (*n* = 344)	*r* = 0.443; *p* < 0.001	*r* = 0.467; *p* < 0.001
Goettingen sentence test (dB SPL) (*n* = 344)	*r* = 0.310; *p* < 0.001	*r* = 0.330; *p* < 0.001
Do you have a problem tolerating sounds because they often seem much too loud? That is, do you often find too loud or hurtful sounds which other people around you find quite comfortable? (*n* = 344)	*r* = –0.025; *p* = 0.644	*r* = 0.167; *p* = 0.002
Do sounds cause you pain or physical discomfort? (yes: *n* = 186; no = 125)	yes: 0.97 ± 1.25; no: 0.82 ± 1.17; *F* = 0.296; *df* = 1308; *p* = 0.645	yes: 2.66 ± 1.55; no: 2.07 ± 1.61; *F* = 1.685; *df* = 1308; *p* = 0.195

*r, Pearson correlations coefficient; F, analysis of covariance; xx ± xx, mean ± sd*.

## Discussion

The study of speech comprehension difficulties in a large sample of tinnitus patients revealed several main findings. First, with a prevalence of about 40%, subjectively-reported speech comprehension deficits are frequent among tinnitus patients. This number substantially increased when patients were asked about speech comprehension in noisy environments (cocktail party situation) where almost 80% of all investigated tinnitus patients reported difficulties. We are aware that there may be a selection bias as the sample comes from a tertiary referral clinic. However, our sample is much larger than previously investigated samples (Huang et al., 2007) and consisted of unselected patients who consecutively presented because of tinnitus in our clinic. Moreover, there are similar reports of high prevalence of speech intelligibility deficits in workers with exposure to noise and tinnitus (Soalheiro et al., 2012). A further aspect that was not considered in this study is the potential influence of tinnitus-related cognitive or attentional impairment on speech comprehension difficulties. Therefore population-based studies and studies involving cognitive testing will be needed for a valid estimation of the prevalence of speech comprehension difficulties among tinnitus patients. Future studies should also consider the use of more specific diagnostic tests for the presence of hyperacusis beyond the screening questions used in this study. In order to identify the relevance of tinnitus in the interplay between hearing loss and speech comprehension, future research should also include people without tinnitus.

Subjectively-reported speech comprehension difficulties were correlated with the Goettingen sentence test scores. The highly-significant correlation between the subjective perception reports and the behavioral audiological measurements with the Goettingen sentence test provides some validation for the speech comprehension-related questions used for this study. On the other hand, the score in the Goettingen sentence test only explained about 12% of the variance (*r*^2^) of the subjective speech comprehension impairment. This suggests that the two measurements reflect different aspects and arguing for the usefulness of both subjective and behavioral assessment of speech comprehension.

As most people with tinnitus have some form of cochlear damage, both hearing impairment and tinnitus can contribute to impaired speech comprehension in tinnitus patients (Tyler and Baker, 1983). Here, we found a high correlation between audiometrically-determined hearing threshold shifts and the subjectively-perceived speech comprehension difficulties. This clearly suggests that the degree of cochlear damage has a significant impact of speech comprehension difficulties—both in general (per question 1) and in “cocktail party” situations (per question 2). On the other hand, both our data and previous studies show that there are also tinnitus patients with normal hearing who complain about speech comprehension deficits (Goldstein and Shulman, 1999; Soalheiro et al., 2012) highlighting the role of non-peripheral modulating factors of speech comprehension deficits.

We also investigated the relationship between co-morbid hyperacusis and speech comprehension difficulties. Answers to the two screening questions for hyperacusis (Schecklmann et al., 2015) were significantly related to speech comprehension in the cocktail party situation, but not to speech comprehension in general. As hyperacusis is presumably due to increased central gain as a consequence of deficient central inhibitory mechanisms (Brotherton et al., 2015), our findings suggest that this inhibitory dysfunction of the auditory pathway has a specific impact on speech comprehension difficulties in the cocktail party situation.

According to current knowledge, a central inhibitory deficit is occurring both in tinnitus and hyperacusis, but is especially pronounced in hyperacusis (Noreña, 2011; Hébert et al., 2013). This central inhibitory deficit seems to be particularly relevant for speech comprehension deficits in noisy environments. One could speculate that speech comprehension in cocktail party situations requires functional inhibitory systems in the neural auditory pathways to actively filter out irrelevant sounds and this function is impaired in tinnitus patients and particularly in tinnitus patients with hyperacusis. This “Central Inhibitory Deficit”—hypothesis would fit with the reported improvement of speech-in-noise perception after successful tinnitus reduction (Barwood et al., 2013; Mertens et al., 2013b).

The medial olivary cochlear system is involved in the auditory efferent system originating in the auditory cortex with connections to the inner and outer hair cells (Hennig et al., 2012). This filter system for peripheral input (Harkrider and Bowers, 2009) takes part in the processing of interaural time differences for sound localization (Myoga et al., 2014), which is necessary for conversation in noisy environment. Moreover, the medial olivary cochlear system has anti-masking functions to adjust cochlear amplification in situations of listening to speech-in-noise (Bidelman and Bhagat, 2015). This might be involved in hyperacusis as shown by decreased distortion-product otoacoustic emissions, as elicited by noise presented to the contralateral ear in patients with tinnitus and low sound level tolerance (Knudson et al., 2014).

Previously, an association between otoacoustic emissions and hyperacusis (but not with hearing in noise) was found (Spyridakou et al., 2012). This study showed elevated hyperacusis scores and reduced otoacoustic emissions of 10 patients with auditory processing disorder but normal hearing in contrast to 12 age-matched controls. Nineteen normal-hearing patients with tinnitus and hyperacusis showed similar performance in speech comprehension in silence, but lower performance in a communication scenario in contrast to 23 normal hearing subjects without hearing complaints (Hennig et al., 2012). In conclusion, a central inhibitory deficit (e.g., an impairment of the medial olivary cochlear system) might represent the common pathophysiologic substrate of tinnitus, hyperacusis, and speech comprehension (in noise) (Hennig et al., 2012).

Electroencephalographic studies have demonstrated that auditory alpha oscillations, which are reduced in tinnitus patients, are critically involved in speech comprehension(Weisz et al., 2011), suggesting that reduced alpha oscillations in the temporal cortex could represent a neuronal correlate of deficient central inhibitory activity in the auditory system, which can manifest as tinnitus and as deficient speech-in-noise comprehension.

In summary, our data suggest that speech comprehension difficulties occur frequently among tinnitus patients and are caused by both peripheral hearing loss and deficient central inhibitory mechanisms. The latter are probably important for speech comprehension difficulties in noisy environments.

The presented work has several implications for clinical management and research. First, our data indicate that speech comprehension difficulties occur frequently among tinnitus patients and should be regularly explored in clinical routine. Second, the proposed screening questions can be used for a simple and easily feasible standardized assessment of speech comprehension difficulties in tinnitus patients. Third, our data also suggest that it would be worthwhile to investigate whether improvement of tinnitus severity and hyperacousis, e.g., by cognitive behavioral therapy, might also improve speech comprehension in noise. Fourth, the effects of hearing aids and specific forms of hearing training should be evaluated in clinical trials.

## Author contributions

VV and BL: conception, acquisition, interpretation, manuscript. PK and FH: acquisition, manuscript. TS and WS: interpretation, analysis, manuscript. PS: acquisition, interpretation, manuscript. TK: conception, interpretation, manuscript. MS: acquisition, interpretation, analysis, manuscript.

### Conflict of interest statement

The authors declare that the research was conducted in the absence of any commercial or financial relationships that could be construed as a potential conflict of interest.

## References

[B1] Araujo T. M. Iório M. C. M. (2016). Effects of sound amplification in self-perception of tinnitus and hearing loss in the elderly. Braz. J. Otorhinolaryngol. 82, 289–296. 10.1016/j.bjorl.2015.05.010 26541231 PMC9444603

[B2] Arweiler-Harbeck D. Janeschik S. Lang S. Bagus H. (2011). Suitability of auditory speech sound evaluation (A§E®) in German cochlear implant patients. Eur. Arch. Otorhinolaryngol. 268, 1259–1266. 10.1007/s00405-011-1505-2 21305312

[B3] Baguley D. McFerran D. Hall D. (2013). Tinnitus. Lancet 382, 1600–1607. 10.1016/S0140-6736(13)60142-7 23827090

[B4] Barwood C. H. Wilson W. J. Malicka A. N. McPherson B. Lloyd D. Munt K.. (2013). The effect of rTMS on auditory processing in adults with chronic, bilateral tinnitus: a placebo-controlled pilot study. Brain Stimul. 6, 752–759. 10.1016/j.brs.2013.01.015 23453932

[B5] Bidelman G. M. Bhagat S. P. (2015). Right-ear advantage drives the link between olivocochlear efferent ‘antimasking’ and speech-in-noise listening benefits. Neuroreport 26, 483–487. 10.1097/WNR.0000000000000376 25919996

[B6] Brotherton H. Plack C. J. Maslin M. Schaette R. Munro K. J. (2015). Pump up the volume: could excessive neural gain explain tinnitus and hyperacusis? Audiol. Neurootol. 20, 273–282. 10.1159/000430459 26139435

[B7] De Ridder D. Elgoyhen A. B. Romo R. Langguth B. (2011). Phantom percepts: tinnitus and pain as persisting aversive memory networks. Proc. Natl. Acad. Sci. U.S.A. 108, 8075–8080. 10.1073/pnas.1018466108 21502503 PMC3100980

[B8] Goebel G. Hiller W. (1994). [The tinnitus questionnaire. A standard instrument for grading the degree of tinnitus. Results of a multicenter study with the tinnitus questionnaire]. HNO 42, 166–172. German. 8175381

[B9] Goldstein B. Shulman A. (1999). Central auditory speech test findings in individuals with subjective idiopathic tinnitus. Int. Tinnitus J. 5, 16–19. 10753411

[B10] Harkrider A. W. Bowers C. D. (2009). Evidence for a cortically mediated release from inhibition in the human cochlea. J. Am. Acad. Audiol. 20, 208–215. 10.3766/jaaa.20.3.7 19927691

[B11] Hébert S. Fournier P. Noreña A. (2013). The auditory sensitivity is increased in tinnitus ears. J. Neurosci. 33, 2356–2364. 10.1523/JNEUROSCI.3461-12.2013 23392665 PMC6619157

[B12] Hennig T. R. Costa M. J. Rossi A. G. Moraes A. B. (2012). Auditory rehabilitation effects on the temporal ordering ability in elderly hearing aids users. J. Soc. Bras. Fonoaudiol. 24, 26–33. 10.1590/S2179-64912012000100006 22460369

[B13] Hiller W. Goebel G. (1992). A psychometric study of complaints in chronic tinnitus. J. Psychosom. Res. 36, 337–348. 1593509 10.1016/0022-3999(92)90070-i

[B14] Hiller W. Goebel G. Rief W. (1994). Reliability of self-rated tinnitus distress and association with psychological symptom patterns. Br. J. Clin. Psychol. 33 (Pt 2), 231–239. 8038742 10.1111/j.2044-8260.1994.tb01117.x

[B15] Hoffmann H. J. Reed G. W. (2004). Epidemiology of tinnitus, in Tinnitus: Theory and Management, ed Snow J. (London: BC Decker), 16–41.

[B16] Huang C. Y. Lee H. H. Chung K. C. Chen H. C. Shen Y. J. Wu J. L. (2007). Relationships among speech perception, self-rated tinnitus loudness and disability in tinnitus patients with normal pure-tone thresholds of hearing. ORL J. Otorhinolaryngol. Relat. Spec. 69, 25–29. 10.1159/000096713 17085949

[B17] Jones G. L. Litovsky R. Y. (2008). Role of masker predictability in the cocktail party problem. J. Acoust. Soc. Am. 124, 3818–3830. 10.1121/1.2996336 19206808 PMC2676623

[B18] Knudson I. M. Shera C. A. Melcher J. R. (2014). Increased contralateral suppression of otoacoustic emissions indicates a hyperresponsive medial olivocochlear system in humans with tinnitus and hyperacusis. J. Neurophysiol. 112, 3197–3208. 10.1152/jn.00576.2014 25231612 PMC4269714

[B19] Kollmeier B. Wesselkamp M. (1997). Development and evaluation of a German sentence test for objective and subjective speech intelligibility assessment. J. Acoust. Soc. Am. 102, 2412–2421. 9348699 10.1121/1.419624

[B20] Landgrebe M. Zeman F. Koller M. Eberl Y. Mohr M. Reiter J.. (2010). The Tinnitus Research Initiative (TRI) database: a new approach for delineation of tinnitus subtypes and generation of predictors for treatment outcome. BMC Med. Inform. Decis. Mak. 10:42. 10.1186/1472-6947-10-42 20682024 PMC2920857

[B21] Langguth B. Goodey R. Azevedo A. Bjorne A. Cacace A. Crocetti A.. (2007). Consensus for tinnitus patient assessment and treatment outcome measurement: Tinnitus Research Initiative meeting, Regensburg, July 2006. Prog. Brain Res. 166, 525–536. 10.1016/S0079-6123(07)66050-6 17956816 PMC4283806

[B22] May-Mederake B. Shehata-Dieler W. (2013). A case study assessing the auditory and speech development of four children implanted with cochlear implants by the chronological age of 12 months. Case Rep. Otolaryngol. 2013:359218. 10.1155/2013/359218 23509653 PMC3590554

[B23] Mertens G. Kleine Punte A. De Ridder D. Van de Heyning P. (2013b). Tinnitus in a single-sided deaf ear reduces speech reception in the nontinnitus ear. Otol. Neurotol. 34, 662–666. 10.1097/MAO.0b013e31828779f0 23640086

[B24] Mertens G. Punte A. K. Van de Heyning P. (2013a). Self-assessment of hearing disabilities in cochlear implant users using the SSQ and the reduced SSQ5 version. Otol. Neurotol. 34, 1622–1629. 10.1097/MAO.0b013e31829ce980 24005165

[B25] Moon I. J. Won J. H. Kang H. W. Kim D. H. An Y. H. Shim H. J. (2015). Influence of tinnitus on auditory spectral and temporal resolution and speech perception in tinnitus patients. J. Neurosci. 35, 14260–14269. 10.1523/JNEUROSCI.5091-14.2015 26490865 PMC6605422

[B26] Myoga M. H. Lehnert S. Leibold C. Felmy F. Grothe B. (2014). Glycinergic inhibition tunes coincidence detection in the auditory brainstem. Nat. Commun. 5, 3790. 10.1038/ncomms4790 24804642 PMC4024823

[B27] Newman C. W. Wharton J. A. Shivapuja B. G. Jacobson G. P. (1994). Relationships among psychoacoustic judgments, speech understanding ability and self-perceived handicap in tinnitus subjects. Audiology 33, 47–60. 8129680 10.3109/00206099409072954

[B28] Noreña A. J. (2011). An integrative model of tinnitus based on a central gain controlling neural sensitivity. Neurosci. Biobehav. Rev. 35, 1089–1109. 10.1016/j.neubiorev.2010.11.003 21094182

[B29] Norena A. Micheyl C. Chéry-Croze S. Collet L. (2002). Psychoacoustic characterization of the tinnitus spectrum: implications for the underlying mechanisms of tinnitus. Audiol. Neurootol. 7, 358–369. 10.1159/000066156 12401967

[B30] Ryu I. S. Ahn J. H. Lim H. W. Joo K. Y. Chung J. W. (2012). Evaluation of masking effects on speech perception in patients with unilateral chronic tinnitus using the hearing in noise test. Otol. Neurotol. 33, 1472–1476. 10.1097/MAO.0b013e31826dbcc4 22996163

[B31] Schaette R. McAlpine D. (2011). Tinnitus with a normal audiogram: physiological evidence for hidden hearing loss and computational model. J. Neurosci. 31, 13452–13457. 10.1523/JNEUROSCI.2156-11.2011 21940438 PMC6623281

[B32] Schecklmann M. Landgrebe M. Langguth B. TRI Database Study Group. (2014). Phenotypic characteristics of hyperacusis in tinnitus. PLoS ONE 9:e86944. 10.1371/journal.pone.0086944 24498000 PMC3908961

[B33] Schecklmann M. Lehner A. Schlee W. Vielsmeier V. Landgrebe M. Langguth B. (2015). Validation of screening questions for hyperacusis in chronic tinnitus. Biomed. Res. Int. 2015:191479. 10.1155/2015/191479 26557654 PMC4628768

[B34] Shore S. E. Roberts L. E. Langguth B. (2016). Maladaptive plasticity in tinnitus–triggers, mechanisms and treatment. Nat. Rev. Neurol. 12, 150–160. 10.1038/nrneurol.2016.12 26868680 PMC4895692

[B35] Soalheiro M. Rocha L. do Vale D. F. Fontes V. Valente D. Teixeira L. R. (2012). Speech recognition index of workers with tinnitus exposed to environmental or occupational noise: a comparative study. J. Occup. Med. Toxicol. 7:26. 10.1186/1745-6673-7-26 23259813 PMC3558344

[B36] Spyridakou C. Luxon L. M. Bamiou D. E. (2012). Patient-reported speech in noise difficulties and hyperacusis symptoms and correlation with test results. Laryngoscope 122, 1609–1614. 10.1002/lary.23337 22565811

[B37] Tyler R. S. Baker L. J. (1983). Difficulties experienced by tinnitus sufferers. J. Speech Hear. Disord. 48, 150–154. 6621006 10.1044/jshd.4802.150

[B38] Vielsmeier V. Lehner A. Strutz J. Steffens T. Kreuzer P. M. Schecklmann M.. (2015). The relevance of the high frequency audiometry in tinnitus patients with normal hearing in conventional pure-tone-audiometry. Biomed. Res. Int. 2015:302515. 10.1155/2015/302515 26583098 PMC4637018

[B39] Weisz N. Hartmann T. Dohrmann K. Schlee W. Norena A. (2006). High-frequency tinnitus without hearing loss does not mean absence of deafferentation. Hear. Res. 222, 108–114. 10.1016/j.heares.2006.09.003 17079102

[B40] Weisz N. Hartmann T. Müller N. Lorenz I. Obleser J. (2011). Alpha rhythms in audition: cognitive and clinical perspectives. Front. Psychol. 2:73. 10.3389/fpsyg.2011.00073 21687444 PMC3110491

